# Effects of Dimenhydrinate on Motor Behavior and Vascular Function: Possible Implications for the Field of Sports

**DOI:** 10.1002/dta.3875

**Published:** 2025-03-06

**Authors:** Jazmín Flores‐Monroy, Diana Ramírez‐Hernández, Diego Lezama‐Martínez, Benjamín Velasco‐Bejarano

**Affiliations:** ^1^ Laboratorio de Farmacología del Miocardio, Facultad de Estudios Superiores Cuautitlán Universidad Nacional Autónoma de México Cuautitlán Izcalli Estado de México Mexico; ^2^ Laboratorio de Química Medicinal Verde, Facultad de Estudios Superiores Cuautitlán Universidad Nacional Autónoma de México Cuautitlán Izcalli Estado de México Mexico

**Keywords:** 8‐chlorotheophylline, dimenhydrinate, diphenhydramine, motor behavior, physical activity, vascular reactivity

## Abstract

Several studies have described the sedative effects of dimenhydrinate (DMH), although others report a stimulant effect on psychomotor functions. Because the first generation of antihistamines was shown to seriously impair cognitive psychomotor and driving performance in healthy volunteers, the aim of our research was to determine the effect of DMH by testing physical activity and cognitive and cardiovascular functions using an animal model to identify a possible stimulatory effect. The study protocol consisted of two phases. The first was designed to analyze the stimulating motor effect of DMH. Four study groups were formed: (1) vehicle (Veh), (2) modafinil (MOD), (3) DMH at 50 mg/kg (DMH‐50), and (4) DMH at 200 mg/kg (DMH‐200). Motor coordination and balance, physical activity, hemodynamics, and nitrous oxide (NO) quantification were performed. In the second phase, we sought to discriminate the compound in DMH that generates the stimulating effect. In this case, the study groups were (1) Veh, (2) MOD, (3) DMH, (4) diphenhydramine (DPH), (5) 8‐chlorotheophylline (8‐Cl‐T), and (6) theophylline (TEO). In this phase, we quantified glucose and insulin levels, behavior, physical activity, blood pressure, and vascular reactivity to phenylephrine and acetylcholine. Findings showed that DMH might improve a motor and physical stimulating effect but also increased NO levels in the lungs. DPH promoted a compulsive‐like behavior that diminished with 8‐Cl‐T. Regarding cardiovascular effects, DMH decreased vascular reactivity to phenylephrine and acetylcholine. Finally, in the DMH formulation, 8‐Cl‐T was identified as the compound responsible for increasing blood pressure and heart rate.

## Graphical abstract

Regarding cardiovascular effects, DMH decreased vascular reactivity to phenylephrine and acetylcholine. We observed that the combination of DPH and 8‐Cl‐T was identified as the responsible for increasing blood pressure and heart rate in the biological model used.

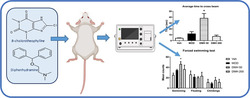

## 1 Introduction

Consuming certain substances has the potential to enhance the performance of athletes during competitions, so it is important to obtain scientific evidence or pharmacological data on the potential of substances, alone or combined with others, to improve an athlete's performance. The use of antihistamines is, of course, common in the population, but they exert distinct effects that have not yet been analyzed in sufficient depth, including their possible ergogenic effect [1]. Dimenhydrinate (DMH) is a drug in the antihistamine family, a theoclate salt composed of diphenhydramine (DPH), an ethanolamine derivative, and 8‐chlorotheophylline (8‐Cl‐T) (Table 1), a chlorinated theophylline (TEO) derivative, in a ratio of 1:1. DMH is used to treat motion sickness and nausea [2]. It has been reported that a single oral dose of DPH hydrochloride (50 mg) does not compromise or improve performance on treadmill tests in physically active subjects [3]. DPH has been available as an antihistamine since 1949. Currently, it is sold over‐the‐counter. It is the drug most commonly prescribed for children as a medication to treat allergies and insomnia. However, DPH has been related to other drugs that generate stimulant effects, specifically in the 1960s when it was taken with methaqualone as a recreative drug—known as “blue velvet”—that had effects similar to those of some stimulants [4].

**TABLE 1:** Chemical structure and description of diphenhydramine (DPH), 2‐[(diphenylmethyl)sulfinyl]acetamide (Modafinil, MOD), 8‐chloro‐1,3‐dimethyl‐7*H*‐purine‐2,6‐dione (8‐ cholorotheophylline, 8‐Cl‐T), and theophylline (TEO).

Chemical structure	Description
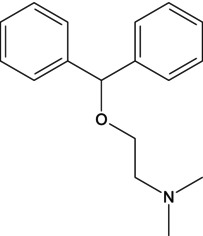	Diphenhydramine is a molecule formed structurally of a diphenyl residue similar to the one present in the Modafinil molecule. An ether present gives a certain polarity due to the oxygen atom.
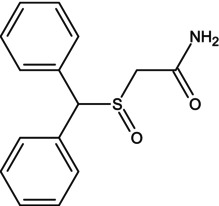	Modafinil is a molecule with biphenyl residue, sulfoxide, and a terminal amide in its structure. The molecule is structurally similar to dimenhydrinate due to the diphenyl residue that both share. A sulfur atom is present in the Modafinil molecule that bonds to the diphenyl structure. In dimenhydrinate, an oxygen atom is found at this distance.
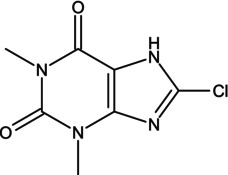	8‐Cholorotheophylline (8‐Cl‐T) is a derivative of theophylline, a compound with a chemical structure similar to its precursor. The only structural modification is the presence of a chlorine atom in eight positions in the theophylline structure. The presence of a more electronegative atom modifies theophylline's pharmacological activity, so it exhibits physiological effects similar to those of caffeine. Log Kow = −0.02
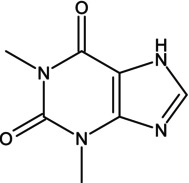	Theophylline is a dimethylxanthine with two methyl groups at positions 1 and 3. It is structurally similar to caffeine. Two heterocyclic rings with nitrogen atoms form its structure.

The component 8‐Cl‐T is a methylxanthine that has been shown to have a potent positive inotropic effect. It is equivalent to TEO in terms of its pharmacological response. A concentration of only 1/15th of nonionized 8‐Cl‐T suffices to obtain the same response as TEO [5]. TEO appears to improve exercise performance [6], because some psychomotor stimulant effects have been described in animal studies after methylxanthine administration [7]. Therefore, the combination of DPH and 8‐Cl‐T in DMH could have stimulant effects on the nervous system, as the drug produces effects related to dopamine, acetylcholine, serotonin, norepinephrine, and opioids [8]. For some time, antihistamines like DMH and DPH have been used for recreational purposes due to toxic effects like thought disorders and visual, auditory, and tactile hallucinations. These symptoms seem to be similar to those of atropine and could be due to anticholinergic effects [8].

In light of the foregoing, the present study was designed to determine the stimulant motor behavior and cardiovascular effect of DMH by testing physical activity and cognitive and vascular reactivity in a biological model to identify possible side effects on motor responses and cardiovascular functions.

## 2 Materials and Methods

### 2.1 Chemicals and Reagents

Modafinil (MOD) was obtained from Lundbeck Mexico SA de CV (Mexico City). DMH was obtained from Toronto Research Chemical Inc. (Toronto, Canada). DPH, 8‐Cl‐T, TEO, and ethanol used were HPLC grade. Acetylcholine, L‐phenylephrine, and VCl_3_ were obtained from Merck, Mexico. The drugs were diluted in physiological saline solution and 70% ethanol before administration at specific concentrations.

### 2.2 Animals

Fifty female Wistar rats (20 weeks old, body weight ~300 g) were obtained from CINVESTAV, Southern Unit (IPN). They received water and rat chow (LabDiet 5001) ad libitum and were housed in acrylic boxes under standard laboratory conditions. All procedures were conducted according to the Federal Regulations for Animal Experimentation and Care (SAGARPA, NOM‐062‐ZOO.1999, Mexico) and the National Institutes of Health Guide for the Care and Use of Laboratory Animals (NIH Publication No. 8023, revised 1978, USA). The experimental protocol was approved by the local Ethics Committee of the FES Cuautitlán, UNAM, under registration code CICUAE‐FESC C19‐13.

### 2.3 Experimental Design

This experiment was divided into two phases: *Phase 1*: 20 female Wistar rats, described above, were distributed in four groups (*n* = 5 for all groups): (1) saline (Veh), (2) Modafinil at 64 mg/kg (MOD), (3) DMH at 50 mg/kg (DMH‐50), and (4) DMH at 200 mg/kg (DMH‐200). All pharmacological treatments were administered *i.p*. 1 h before the experiments. The beam‐walking and forced swimming tests were performed. Behavioral and physical activity assessments were conducted at 8:00 a.m. under the following conditions: white noise background, reduced light, and room temperature around 24 °C. After the behavioral and physical activity tests, the rats were euthanized, cardiac catheterization was performed, and serum and tissue (aorta, heart, lung) samples were obtained to quantify nitric oxide (NO) content.

*Phase 2*: Thirty female Wistar rats, described above, were divided into six groups (*n* = 5 for all groups): (1) saline (Veh), (2) Modafinil at 64 mg/kg (MOD), (3) dimenhydrinate at 60 mg/kg (DMH), (4) diphenhydramine at 32.4 mg/kg (DPH), (5) 8‐chlorotheophylline at 27.6 mg/kg (8‐Cl‐T), and (6) theophylline at 27.6 mg/kg (TEO). As in phase 1, all pharmacological treatments were administered via *i.p*. 1 h before the experiments. The dosages of DPH, 8‐Cl‐T, and TEO were determined based on the equipotent dose of DMH. Before and after treatment administration, arterial blood pressure was measured and the marble burying and forced swimming tests were performed. At that point, the animals were euthanized using sodium pentobarbital (50 mg/kg*, i.p*.) to obtain serum and the thoracic aortic tissue samples required to perform the concentration‐response curves to acetylcholine (ACh) and phenylephrine in the aortic rings and quantify serum glucose and insulin levels.

### 2.4 Behavioral Motor Function

The beam‐walking test evaluates motor activity using an elevated 60‐cm wooden beam (3 × 2.5 × 120 cm) to measure latencies to crossing. Each trial lasted a maximum of 60 s [9, 10]. All groups were trained previously on the apparatus for 30 min over 5 days. The rats were allowed to become habituated to the experimental room for 60 min before training and experiments. Error scores were not registered.

### 2.5 Forced Swimming Test

After the beam‐walking test, the rats' physical activity was assessed on the forced swimming test. The animals were trained for 15 min, 24 h before evaluation, and allowed to stay in the evaluation room for 1 day before testing. The rats were placed in a container filled with water. They were unable to escape, and their rear paws could not touch the bottom. The first 5‐min test was performed 24 h after exposure to the device. One count was scored for each 5 s of continuous swimming or floating. Floating was characterized by a lack of movement apart from that necessary for the rat to keep its head above water. Climbing counts characterized by vigorous paddling were also scored. The animals were not driven to exhaustion because only the effect on motor function was to be observed, as has been reported in other studies [11]. All sessions were videotaped for further analysis. After these sessions, the animals were dried and prepared for cardiac catheterization.

### 2.6 Marble Burying Test

To conduct the marble burying test, standard polycarbonate rat cages measuring 30.5 × 30.5 × 48 cm with fitted filter‐top covers were used. Each cage was filled with fresh, unscented mouse bedding to a depth of 5 cm to form a level surface. Toy glass marbles 15 mm in diameter and weighing 5.2 g were placed gently on the bedding in five rows, each with four marbles. One rat was placed in the cage. Food and water were withheld during testing. The rat was left undisturbed for 15 min, then returned to its home cage. The number of marbles buried was counted [12].

### 2.7 Cardiac Catheterization

The rats were anesthetized by a 35‐mg/kg solution of sodium pentobarbital *i.p*.; then, tracheotomies were performed, and the right carotid artery was cannulated with a previously heparinized PE‐50 catheter connected to a pressure transducer (Biopaq Systems, Santa Barbara, California). The catheter was inserted into the left ventricle to measure left ventricular systolic pressure (LVSP), left ventricular diastolic pressure (LVDP), the maximum range of isovolumetric pressure (+dP/dtmax), decay (−dP/dtmax), and heart rate (HR) [13].

### 2.8 Vascular Reactivity

The aortas were removed and placed in fresh Krebs–Henseleit solution containing 118 mM NaCl, 4.7 mM KCl, 1.2 mM KH_2_PO_4_, 1.2 mM MgSO_4_‐7H_2_O, 2.5 mM CaCl_2_‐2H_2_O, 25 mM NaHCO_3_, 11.7 mM dextrose, 0.026 mM calcium, and disodium EDTA. Tissues were oxygenated continuously with a 95% O_2_/5% CO_2_ mixture throughout the procedure. Connective, adipose, and adherent tissues were removed from the aorta and sectioned into 3‐mm‐long rings. Each ring was transferred to a 10‐mL tissue chamber containing Krebs–Henseleit solution at 37°C and pH 7.4, maintaining oxygenation. The rings were suspended between two Nubryte wire hooks to measure the tension. One hook was fixed to the bottom of the chamber, the other to a force transducer (BIOPAC TSD125C) connected to a BIOPAC MP100A‐ce system (BIOPAC Systems Inc., Santa Barbara, California). Measurements were made with the Acknowledge 3.0 software. The initial tension applied to each aortic ring was 3 g. The vasoconstrictor effect of phenylephrine was tested in a concentration range of 1 × 10^−12^ to 1 × 10^−6^ M, followed by a concentration‐response curve for acetylcholine from 1 × 10^−12^ to 1 × 10^−6^ M.

### 2.9 Nitric Oxide Quantification

Quantification of nitric oxide (NO) by the Griess reaction: Blood samples were taken to obtain serum from the study groups and quantify the products of NO metabolism utilizing VCl_3_ (at 0.8% diluted in 10% HCl). The proteins in the serum samples were precipitated by adding 100 μL of serum and 200 μL of an ethanol:water (7:1) solution, followed by centrifugation at 14,500 rpm for 20 min. After that, 100 μL of VCl_3_ and 50 μL of sulfinamide (2% diluted in HCl 10%) were added to 100 μL of the supernatant in a microtiter plate (Illinois, USA).

### 2.10 Glucose and Insulin Quantification

Serum glucose levels were assessed after treatment in all experimental groups using a spectrophotometer. Measurements were taken following the manufacturer's guidelines (Wiener Lab, glycemia AA enzyme). The Millipore ELISA kit (RAB0904, Sigma‐Aldrich, lot #0712I0743) was used according to the supplier's instructions.

### 2.11 EC_50_ and Emax Values

These data were obtained from the results of the dose–response curves for angiotensin II, phenylephrine, and acetylcholine in the GraphPad prism 8.0 software.

### 2.12 Statistical Analysis

Results (*n* = 5 per group) are expressed as means ± standard error (SEM). The concentration–response curves were analyzed by a two‐way ANOVA and multiple comparisons using Tukey's test. Differences in the data were determined by a one‐way analysis of variance and Student Newman–Keuls post hoc analysis. Statistical significance was set at *p* < 0.05. All statistical analyses were performed using the GraphPad prism 8 software.

## 3 Results

### 3.1 Phase 1

The objective was to determine if high doses of DMH had similar effects to those of MOD on the stimulation of motor and hemodynamic cardiac activity. This was proposed due to the similarities in the chemical structure of MOD and DPH, the latter being the pharmacophore group of DMH (Table 1).

**FIGURE 1:**
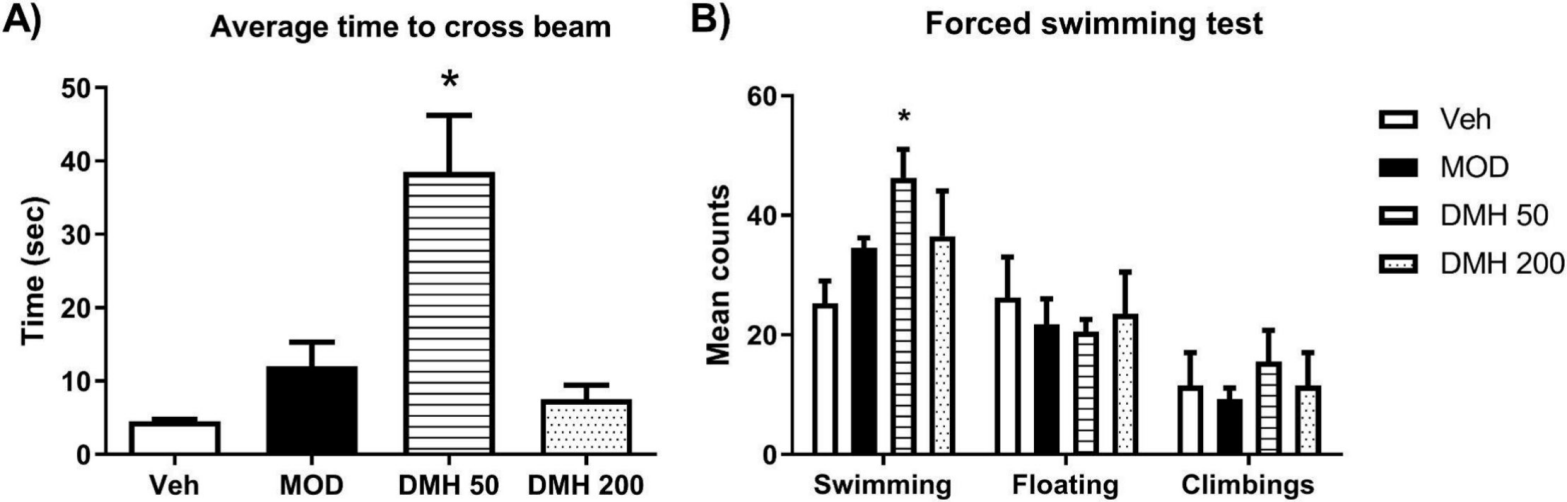
Beam‐walking and forced swimming test results of rats treated with MOD, DMH‐50, DMH‐200, and Veh (SSF). (A) Average time to cross the beam for all groups. DMH‐50 increased this time compared with Veh, *p < 0.05 versus Veh. (B) Forced swimming test. DMH‐50 increased its mean counts of swimming compared with Veh. Results are reported as means ± SEM, *n* = 5 rats per group. *p < 0.05 versus Veh.

Figure 1A depicts the effects of DMH and MOD on the rats during the beam‐walking test. The DMH‐50 group increased its latency to crossing the beam, but the MOD and DMH‐200 groups showed no modification in their responses compared with the Veh group. Figure 1B shows the effects of DMH and MOD on the rats during the forced swimming test. In this case, the DMH‐50 group increased its swimming counts compared with Veh. In contrast, the MOD and DMH‐200 groups showed no change in their results compared with Veh.

Table 2 shows the results of the cardiac catheterization of the rats after pharmacological treatment outlined above. The MOD and DMH‐200 groups showed significant increases in LVSP compared with Veh. No significant differences were found for the other parameters.

**TABLE 2:** Hemodynamic parameters observed in the Wistar rats treated with MOD, DMH‐50, and DMH‐200.

Group	SBP (mmHg)	DBP (mmHg)	HR(b/min)	LVSP (mmHg)	LVDP (mmHg)	+dP/dt (mmHg/s)	−dP/dt (mmHg/s)
Veh	135 ± 22	96 ± 21	428 ± 32	149 ± 17	1 ± 1	2648 ± 289	3128 ± 531
MOD	175 ± 20	141 ± 26	319 ± 18	225 ± 29 *	0	4513 ± 504	4431 ± 575
DMH‐50	133 ± 5	93 ± 7	349 ± 94	119 ± 11	0	2530 ± 677	2524 ± 575
DMH‐200	170 ± 4	133 ± 5	347 ± 27	208 ± 10 *	4 ± 3	4158 ± 707	3687 ± 431

*Note:* Values are means ± standard error, *n* = 5 per group. One‐way ANOVA followed by a Student Newman–Keuls test.

Abbreviations: +dP/dt, maximum isovolumetric pressure; −dP/dt, maximum range of isovolumetric pressure decay; HR, heart rate; PAD, diastolic blood pressure; PAS, systolic blood pressure; PDVI, left ventricular diastolic pressure; PSVI, left ventricular systolic pressure.

* *p* < 0.05 versus Veh.

**FIGURE 2:**
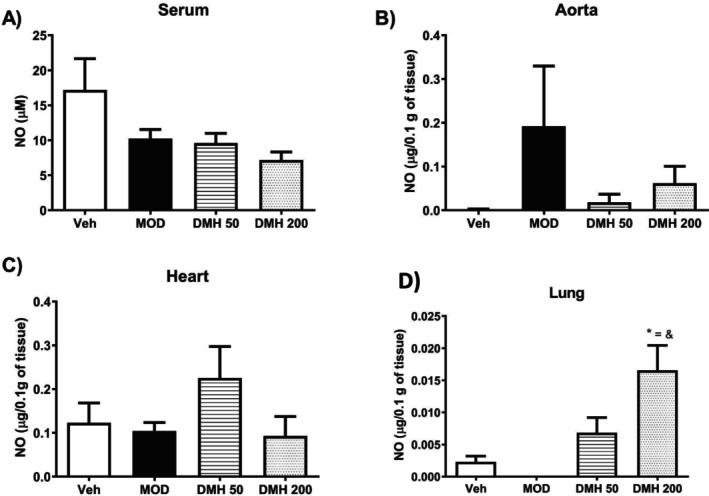
NO levels in the serum, aorta, heart, and lung tissues of rats treated with MOD, DMH‐50, DMH‐200, and Veh. (A) Seric NO levels. (B) Aorta NO levels. (C) Heart NO levels; (D) Lung NO levels. The DMH‐200 group increased NO levels compared with Veh, MOD, and DMH‐50, *p < 0.05 versus Veh; =*p* < 0.05 versus MOD; &*p* < 0.05 versus DMH‐50. Results reported as means ± SEM, *n* = 5 rats per group. One‐way ANOVA followed by a Student Newman–Keuls test.

In this phase, NO concentrations were determined to evaluate whether DMH had an effect on the production of this vasodilator compound in distinct tissue types (Figure 2). A significant increase was found in lung NO levels in the DMH‐200 group versus Veh, MOD, and DMH‐50, as shown in Figure 2C. NO was also quantified in the serum, aorta, and heart tissues, but no differences were observed (Figures 2A,B,D).

### Phase 2

**FIGURE 3:**
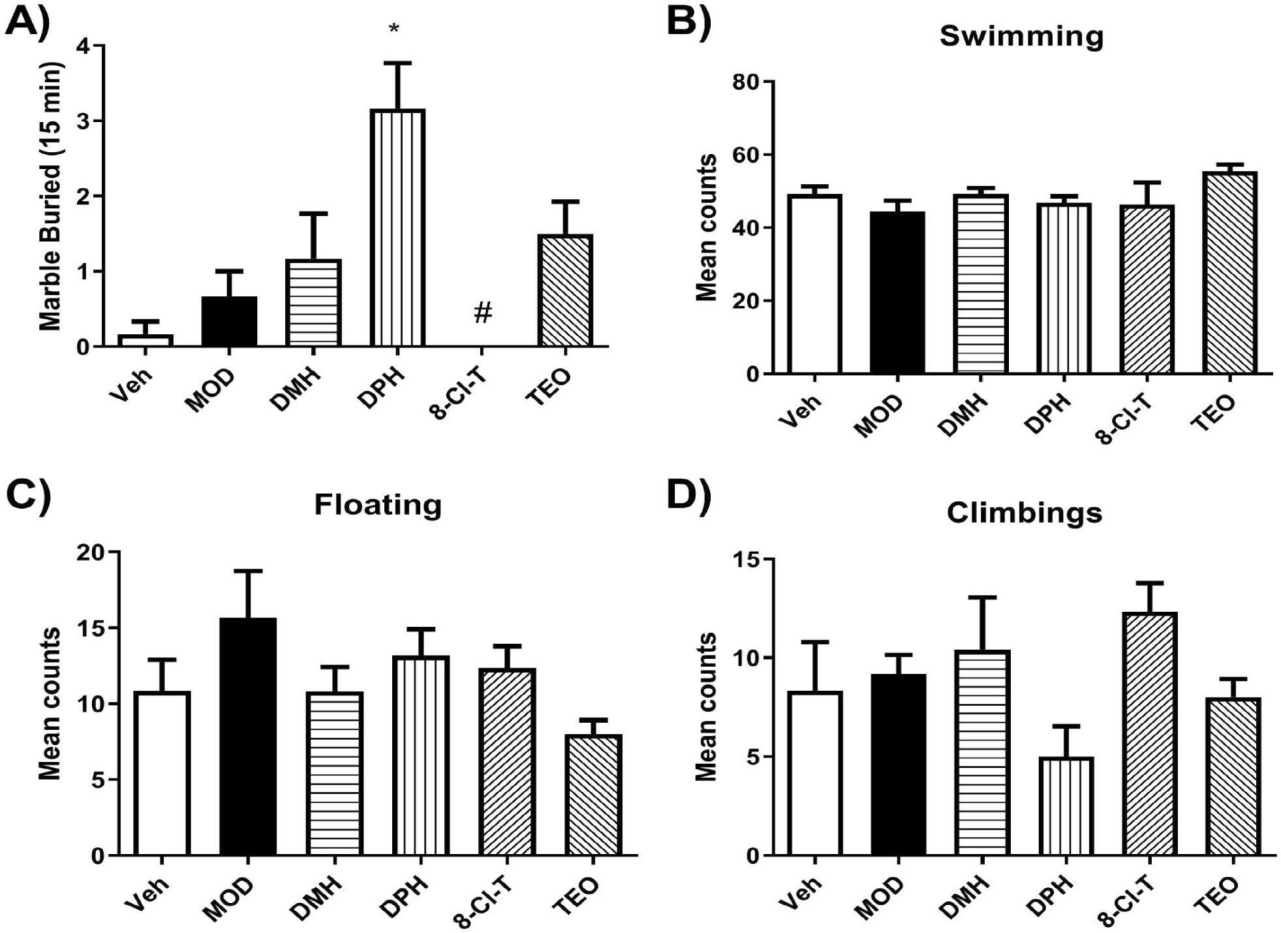
Results of the marble buried and forced swimming tests for rats treated with MOD, DMH, DPH, 8‐Cl‐T, TEO, and Veh. (A) Marbles buried in all groups. DPH increased its compulsive‐like behavior compared with Veh, *p < 0.05 versus Veh. The 8‐Cl‐T group showed a decrease compared with DPH, #*p* < 0.05 versus DPH. (B) Mean counts of swimming. (C) Mean counts of floating. (D) Mean counts of climbing. Results are reported as mean values ± SEM from *n* = 5 rats per group. One‐way ANOVA followed by a Student Newman–Keuls test.

After the first experiments and data analysis, we set out to determine the compound that generated the greatest stimulating effect on DMH (Table 1). A marble burying test was performed to assess behavior (Figure 3). Findings showed an increase in compulsive‐like behavior in the DPH group compared with Veh. In contrast, the 8‐Cl‐T group showed a decrease compared with DPH (Figure 3A). For physical activity, we evaluated swimming time, floating time, and the number of climbings in the water but found no significant between‐group changes (Figure 3B,C,D).

**FIGURE 4:**
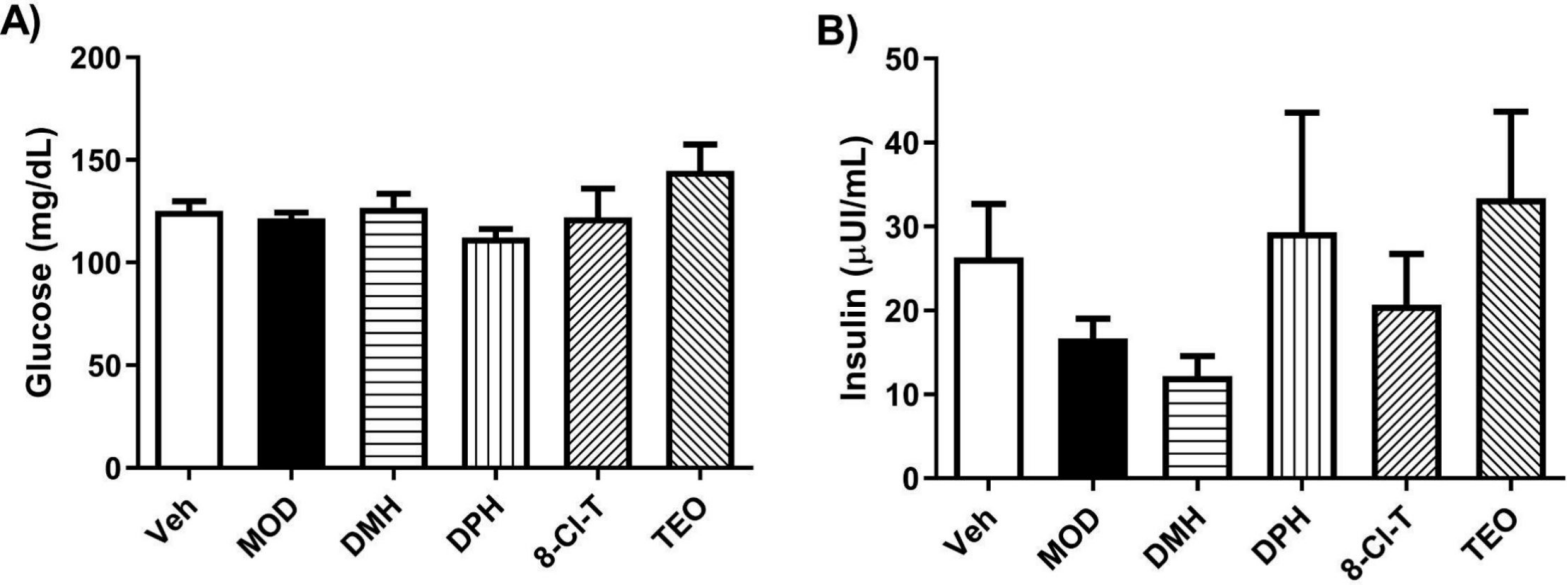
Glucose and insulin levels after administration of MOD, DMH, DPH, 8‐Cl‐T, and TEO. (A) Glucose levels. (B) Insulin levels. Results are reported as means ± SEM, *n* = 5 rats per group. A one‐way ANOVA was performed.

Due to the increase in physical activity in the DMH groups observed in Phase 1, we decided to determine if that result could be related to the increase in glucose blood disposal. Therefore, we measured glucose and insulin levels in all groups (Figure 4A,B). However, no significant changes were observed.

To evaluate the impact of MOD, DMH, and the components of DMH (8‐Cl‐T, DPH) on blood pressure and heart rate (HR), these parameters were measured before and after treatment administration (Table 3). A significant decrease in systolic blood pressure (SBP) was observed in the Veh group after treatment, while in the DMH group, post‐treatment increases in SBP, mean blood pressure (MBP), and HR were verified. All parameters increased in this group after administration of 8‐Cl‐T, although no changes were observed in MOD, DPH, or TEO.

**TABLE 3:** Blood pressure values, measured by the tail cuff model.

Group	SBP (mmHg)		DBP (mmHg)		MBP (mmHg)		HR (beat/min)	
	Before	After	Before	After	Before	After	Before	After
Vehicle	125 ± 2	118 ± 1*	82 ± 1	81 ± 1	97 ± 1	94 ± 1	375 ± 7	371 ± 5
MOD	117 ± 2	122 ± 3	82 ± 1	81 ± 2	93 ± 1	95 ± 2	357 ± 8	377 ± 8
DMH	114 ± 6	122 ± 8*	82 ± 5	85 ± 5	93 ± 5	97 ± 6*	366 ± 2	382 ± 3*
DPH	118 ± 1	118 ± 2	81 ± 1	84 ± 1	97 ± 1	95 ± 1	371 ± 5	376 ± 6
8‐Cl‐T	110 ± 3	128 ± 2*	80 ± 1	88 ± 1*	81 ± 9	101 ± 1*	339 ± 5	375 ± 4*
TEO	125 ± 1	122 ± 3	81 ± 1	80 ± 2	97 ± 1	91 ± 2	375 ± 7	387 ± 5

*Note:* Values are means ± standard error, *n* = 5 rats per group. One‐way ANOVA followed by a Student Newman–Keuls test.

Abbreviations: HR, heart rate; DBP, diastolic blood pressure; MBP, medium blood pressure; SBP, systolic blood pressure.

* *p* < 0.05 versus Veh.

**FIGURE 5:**
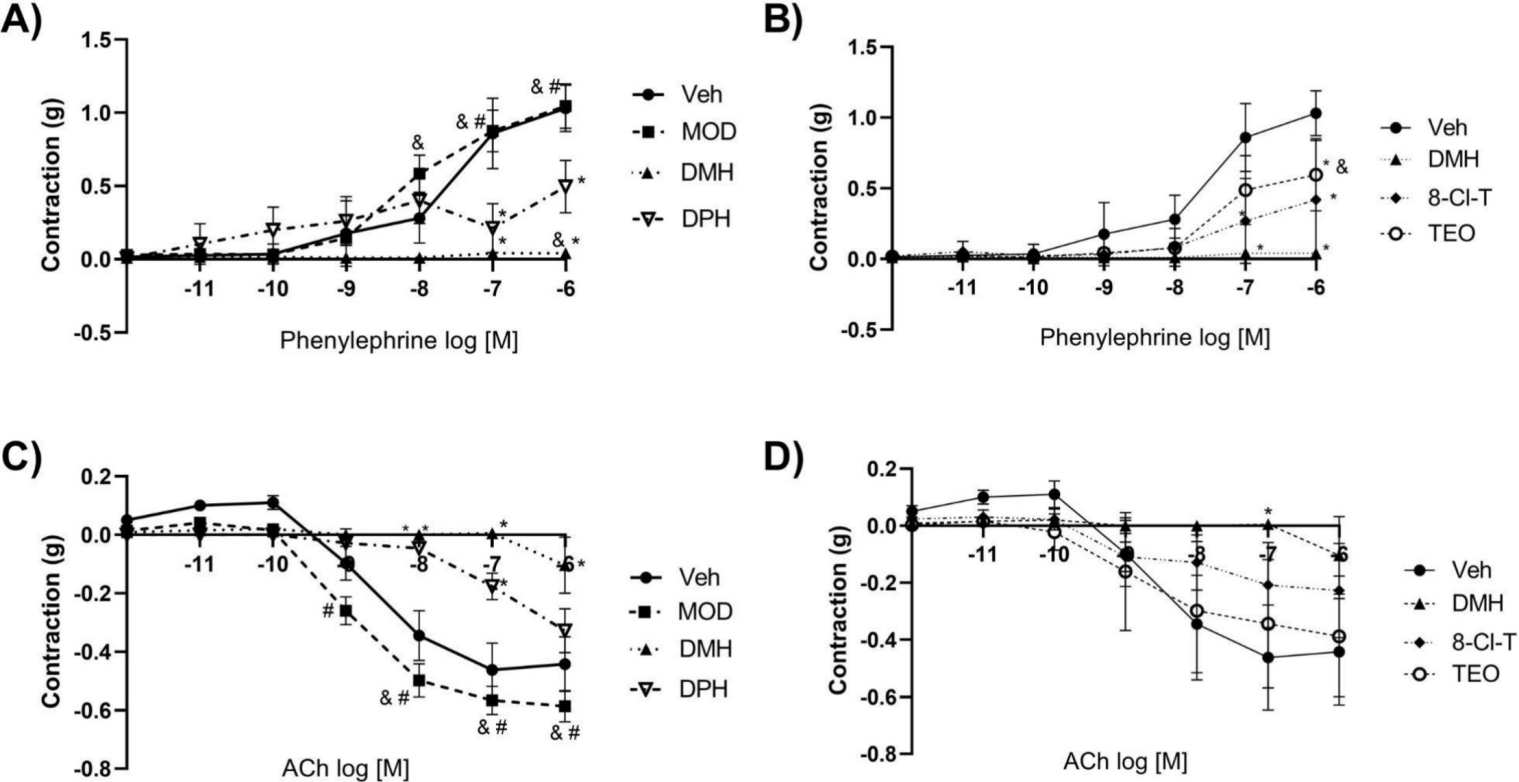
Vascular response to ACh and phenylephrine in the aortic rings from the experimental groups. (A) Vascular reactivity to phenylephrine in rats. DMH and DPH showed significant decreases in vascular responses to phenylephrine compared with Veh (*p < 0.0001 vs. Veh). MOD showed greater vascular reactivity than DMH and DPH (#*p* < 0.0001 vs. DPH; &*p* < 0.0001 vs. DMH). (B) Vascular reactivity to phenylephrine in the rats with Veh, DMH, 8‐Cl‐T, and TEO pre‐treatment. The 8‐Cl‐T and TEO groups had lower vascular reactivity to phenylephrine than Veh (*p < 0.0001; *p = 0.0499, respectively). TEO showed greater vascular reactivity than DMH (&*p* = 0.0364). (C) Vascular reactivity to ACh in the rats after Veh, MOD, DMH, and DPH pre‐treatment. DMH and DPH showed a significant decrease in vascular response to ACh compared with Veh (*p < 0.0001 vs. Veh). MOD had a larger vascular response than DMH and DPH (#*p* < 0.0001 vs. DPH; &*p* < 0.0001 vs. DMH). (D) Vascular reactivity to ACh in the rats after Veh, DMH, 8‐Cl‐T, and TEO pre‐treatment. DMH showed a significant decrease in vascular response to ACh compared with Veh (*p = 0.0114 vs. Veh).

Finally, we assessed the concentration–response curves to ACh and phenylephrine to determine if DMH showed a vascular response in the first stage of the study (Figure 5A,C). In the second phase, vascular reactivity was measured to assess if the components of DMH had an effect on the autonomic nervous system (Figure 5B,D). We found that MOD pre‐treatment did not change the vascular response to ACh or phenylephrine compared with Veh (Figure 5A,C), but that DMH and DPH had lower vascular reactivity to phenylephrine and ACh compared with Veh and MOD. In addition, DMH significantly decreased vascular contraction to phenylephrine compared with DPH. To evaluate the components of DMH, we repeated these concentration‐response curves, finding that 8‐Cl‐T and TEO had decreased vascular contraction to phenylephrine compared with Veh, although no significant differences were found between 8‐Cl‐T and TEO compared with Veh (Figure 5B,D).

Table 4 shows the changes observed in response to the drug treatments on the maximum effect and EC_50_ to phenylephrine and acetylcholine. DMH and 8‐Cl‐T reduced the maximal effect and increased EC_50_ to phenylephrine. In contrast, DMH diminished the effect of acetylcholine, while DPH did not modify it. However, both treatments increased EC_50_ to acetylcholine.

**TABLE 4:** Maximum effect (Emax) and half maximal effective concentration (EC_50_) to phenylephrine and acetylcholine in all groups.

Group	Phenylephrine		Acetylcholine	
	Emax	EC_50_ (nM)	Emax	EC_50_ (nM)
Vehicle	1.06 ± 0.07	28.60 ± 0.16	−0.45 ± 0.04	2.14 ± 0.22
MOD	1.00 ± 0.03	8.06 ± 0.08*	−0.57 ± 0.02	1.14 ± 0.12
DMH	0.04 ± 0.01*	44.5 ± 0.64*	0.10 ± 0.06*	316 ± 2.28*
DPH	0.35 ± 0.04*	0.10 ± 0.47*	−0.36 ± 0.04	105 ± 0.22*
8‐Cl‐T	0.44 ± 0.09*	74.6 ± 0.42*	−0.19 ± 0.02*	0.93 ± 0.3
TEO	0.64 ± 0.06*	43.7 ± 0.22*	−0.36 ± 0.04	0.13 ± 0.08

*Note:* Values are means ± standard error, *n* = 5 rats per group. One‐way ANOVA followed by a Student Newman–Keuls test.

Abbreviations: EC_50_, half maximal effective concentration; Emax, maximal effect.

* *p* < 0.05 versus Veh.

## 4 Discussion

Our results show that DMH increased the behavioral motor function, had a physical stimulating effect, and increased NO lung levels. MOD and DMH (200 mg/kg) increased LVSP. The findings from Phase 1 suggest that DMH improved exercise performance. DPH, meanwhile, promoted compulsive‐like behavior, although 8‐Cl‐T reduced it. The two compounds together as a DMH formulation did not modify this parameter. Regarding cardiovascular effects, DMH decreased vascular reactivity to phenylephrine. Interestingly, DPH and 8‐Cl‐T showed an additive effect on decreasing aortic contraction to phenylephrine. DMH reduced the vascular response to acetylcholine in an effect that can be attributed to the DPH compound, because 8‐Cl‐T did not generate any significant change. In another result, 8‐Cl‐T in the DMH formulation was the compound responsible for increasing blood pressure and HR. Finally, glucose and insulin levels were not involved in the effects of DMH. According to these results, DMH may antagonize the autonomic nervous system in the aorta.

The increased latency on the beam‐walking test under DMH (50 mg/kg) treatment may be associated with the 8‐Cl‐T, because it has been reported that DMH modifies various neurotransmitters, some of which are also influenced by 8‐Cl‐T; hence, this could be related to diverse behavioral effects [7]. The behavioral effects of this agent are attributed primarily to its ability to block adenosine receptors. 8‐Cl‐T produces excitation by blocking the adenosine receptors that inhibit neural firing [14]. In addition, in the forced swimming model, DMH increased swimming time at a dose of 50 mg/kg, but not at 200 mg/kg. It is interesting to note that in Phase 2, no significant changes were observed at the 60‐mg/kg dose, suggesting that the effects of DMH on swimming training could be dose‐dependent. However, to the best of our knowledge, no previous research has studied the effect of DMH in relation to aerobic physical exercise. In this regard, it has been shown that a single oral dose of DPH hydrochloride (50 mg/kg) does not improve performance on exercise tests in physically active subjects [1]. This agrees with our hypothesis that 8‐Cl‐T is the element responsible for the stimulant effects of DMH.

Given the stimulant effects found in the DMH group, we decided to determine if they were related to improved glucose metabolism or an overproduction of NO, but no significant changes were observed in glucose/insulin levels. NO did increase, but only at high doses of DMH (200 mg/kg) in the lungs. In accordance with our results, Goirand et al.’s report on a rat‐perfused isolated lung preparation stated that L‐NAME (an eNOS inhibitor) inhibited 30% of the relaxation induced by TEO [15], suggesting an NO‐dependent mechanism.

In other results, the increases in LVSP, SBP, DBP, MBP, and HR due to DMH were observed under 8‐Cl‐T treatment, but not DPH. This suggests that the inotropic effects observed with DMH could be due to its 8‐Cl‐T component. It is well known that methylxanthines like 8‐Cl‐T increase AMPc levels by blocking PDE, thus increasing calcium influx. This leads us to propose that this could be related to the inotropic effect observed herein [16]. In this regard, there are reports in healthy human subjects that oral administration of DMH increases the HR response to baroreceptor unloading, although without modifying resting muscle sympathetic nerve activity or sympathetic baroreflexes [17]. Among the cardiovascular manifestations observed to date, arrhythmic complications and conduction abnormalities manifested as a wide QRS complex, bundle branch blocking, and a prolonged QT interval induced by DMH and DPH at high doses are visible on electrocardiograms [18, 19].

A contribution of this study was identifying the vascular effects of DMH and its components. We found that DMH treatment reduced the efficacy and potency to phenylephrine of aortic rings, suggesting that 8‐Cl‐T may be the element responsible for this effect because it showed a two‐fold increase of EC_50_. In concordance with this [20], Graf et al. (1995) reported that methylxanthines reduce adrenergic receptor expression in smooth vascular muscle cells in vitro. In contrast, the vasodilatory effect of acetylcholine decreased with DMH and 8‐Cl‐T administration, while methylxanthines have been reported to promote vascular relaxation by reducing intracellular calcium concentrations. It is important to mention, however, that each methylxanthine has distinct effects [21]. In addition, DMH decreased the potency to acetylcholine. The compound responsible for this effect could be DPH, because it increased EC_50_ by a factor of 50‐fold, possibly because first‐generation antihistamines like DPH are antimuscarinic agents [22].

The results presented in this paper concur with Montgomery et al., who affirmed that it is necessary to understand the effects of antihistaminic‐like DMH by studying its side effects, pharmacodynamics, and impact on performance exercise to determine the correct use of these drugs [1].

## 5 Conclusions

In DMH formulation, both 8‐Cl‐T and DPH showed biological activity. DPH increased compulsive behavior but decreased potency to acetylcholine, while 8‐Cl‐T inhibited compulsive behavior, blood pressure, and heart rate, but decreased potency to phenylephrine. DMH might improve physical performance by increasing cardiac function and lung NO levels. At the vascular level, it decreased the response to phenylephrine and acetylcholine. Therefore, the effects of DMH represent the sum total of the biological activity of its various components.

## Conflicts of Interest

The authors declare no conflicts of interest.

## Data Availability

The data that support the findings of this study are available from the corresponding author upon reasonable request.

## References

[1] L. C. Montgomery and P. A. Deuster , “Effects of Antihistamine Medications on Exercise Performance. Implications for Sportspeople,” Sports Medicine 15, no. 3 (1993): 179–195, 10.2165/00007256-199315030-00004. 7680815

[2] A. Bahji , J. Smith , M. Danilewitz , D. Crockford , N. El‐Guebaly , and H. Stuart , “Towards Competency‐Based Medical Education in Addictions Psychiatry: A Systematic Review,” Canadian Medical Education Journal 12, no. 3 (2021): 126–141, 10.36834/cmej.69739. PMC8263022 34249198

[3] L. C. Montgomery and P. A. Deuster , “Ingestion of an Antihistamine Does Not Affect Exercise Performance,” Medicine and Science in Sports and Exercise 24, no. 3 (1992): 383–388. 1549034

[4] M. R. Wallace and E. Allen , “Recovery after Massive Overdose of Diphenhydramine and Methaqualone,” Lancet 2, no. 7580 (1968): 1247–1248, 10.1016/s0140-6736(68)91735-2. 4177229

[5] A. P. Hardman , “American Health Programme,” Medical World 96 (1962): 336–340. 13904628

[6] M. Kennedy , “Effects of Theophylline and Theobromine on Exercise Performance and Implications for Competition Sport: A Systematic Review,” Drug Testing and Analysis 13, no. 1 (2021): 36–43, 10.1002/dta.2970. 33188564

[7] A. G. Halpert , M. C. Olmstead , and R. J. Beninger , “Mechanisms and Abuse Liability of the Anti‐Histamine Dimenhydrinate,” Neuroscience and Biobehavioral Reviews 26, no. 1 (2002): 61–67, 10.1016/S0149-7634(01)00038-0. 11835984

[8] D. F. Craig and C. S. Mellor , “Dimenhydrinate Dependence and Withdrawal,” CMAJ 142, no. 9 (1990): 970–973. 2328468 PMC1451752

[9] R. J. Carter , J. Morton , and S. B. Dunnett , “Motor Coordination and Balance in Rodents,” Current Protocols in Neuroscience 15, no. 1 (2001): 8–12. 10.1002/0471142301.ns0812s15 18428540

[10] T. N. Luong , H. J. Carlisle , A. Southwell , and P. H. Patterson , “Assessment of Motor Balance and Coordination in Mice Using the Balance Beam,” Journal of Visualized Experiments 10, no. 49 (2011): 2376. 10.3791/2376 PMC3197288 21445033

[11] O. V. Bogdanova , S. Kanekar , K. E. D'Anci , and P. F. Renshaw , “Factors Influencing Behavior in the Forced Swim Test,” Physiology & Behavior 118 (2013): 227–239, 10.1016/j.physbeh.2013.05.012. 23685235 PMC5609482

[12] B. W. Jenkins , C. F. Moore , D. Covey , et al., “Evaluating Potential Anxiolytic Effects of Minor Cannabinoids and Terpenes After Acute and Chronic Oral Administration in Rats,” Cannabis and Cannabinoid Research 8, no. S1 (2023): S11–S24, 10.1089/can.2023.0083. 37721993

[13] H. G. Zimmer , W. Zierhut , R. C. Seesko , and A. E. Varekamp , “Right Heart Catheterization in Rats With Pulmonary Hypertension and Right Ventricular Hypertrophy,” Basic Research in Cardiology 83, no. 1 (1988): 48–57, 10.1007/BF01907104. 2454097

[14] J. Bergman and R. D. Spealman , “Behavioral Effects of Histamine H1 Antagonists: Comparison With Other Drugs and Modification by Haloperidol,” Journal of Pharmacology and Experimental Therapeutics 245, no. 2 (1988): 471–478, 10.1016/S0022-3565(25)22640-4. 2896793

[15] F. Goirand , M. Bardou , J. Dumas , L. Rochette , and M. Dumas , “Effects of Phosphodiesterase Inhibitors on Hypoxic Pulmonary Vasoconstriction. Influence of K(+) Channels and Nitric Oxide,” European Journal of Pharmacology 417, no. 1–2 (2001): 141–148. 11301069 10.1016/s0014-2999(01)00900-1

[16] T. Zou , T. Wang , F. Zhen , X. He , S. Dong , and H. Zhang , “Exogenous PDE5 Expression Rescues Photoreceptors in,” Current Medicinal Chemistry 29, no. 40 (2022): 6115–6124, 10.2174/0929867329666220216111952. 35170405

[17] J. R. Carter and C. A. Ray , “Effect of Dimenhydrinate on Autonomic Activity in Humans,” Clinical Autonomic Research 17, no. 3 (2007): 186–192, 10.1007/s10286-007-0417-0. 17530457

[18] M. Farrell , M. Heinrichs , and J. A. Tilelli , “Response of Life Threatening Dimenhydrinate Intoxication to Sodium Bicarbonate Administration,” Journal of Toxicology. Clinical Toxicology 29, no. 4 (1991): 527–535. 1660938 10.3109/15563659109025751

[19] H. E. Hestand and D. W. Teske , “Diphenhydramine Hydrochloride Intoxication,” Journal of Pediatrics 90, no. 6 (1977): 1017–1018, 10.1016/S0022-3476(77)80586-6. 859051

[20] K. A. Jacobson , K. L. Kirk , W. L. Padgett , and J. W. Daly , “Functionalized Congeners of 1,3‐Dialkylxanthines: Preparation of Analogues With High Affinity for Adenosine Receptors,” Journal of Medicinal Chemistry 28, no. 9 (1985): 1334–1340, 10.1021/jm00147a038. 2993622 PMC3468300

[21] J. P. Monteiro , M. G. Alves , P. F. Oliveira , and B. M. Silva , “Structure‐Bioactivity Relationships of Methylxanthines: Trying to Make Sense of all the Promises and the Drawbacks,” Molecules 21, no. 8 (2016): 974, 10.3390/molecules21080974. 27472311 PMC6273298

[22] H. Liu and J. M. Farley , “Effects of First and Second Generation Antihistamines on Muscarinic Induced Mucus Gland Cell ion Transport,” BMC Pharmacology 5 (2005): 8, 10.1186/1471-2210-5-8. 15790419 PMC1079883

